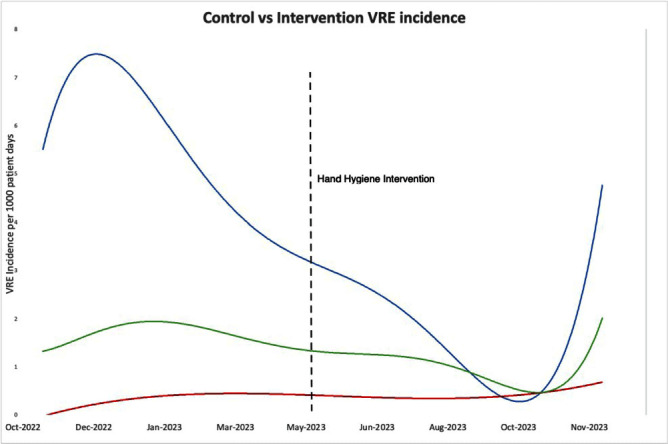# Empowering Patient Hand Hygiene and Reducing Infection in the Oncology Population

**DOI:** 10.1017/ash.2024.249

**Published:** 2024-09-16

**Authors:** Erica LeBlanc, Selasie Ametorwo, Kelsey Houston, Jessica Kociper, Susy Hota, Alon Vaisman

**Affiliations:** University Health Network; UHN IPAC Team; Infection Prevention and Control, University Health Network

## Abstract

**Background:** Significant focus has been placed on healthcare worker hand hygiene, but little attention is has been assigned to the role of patient hand hygiene (HH) in reducing hospital acquired infections. Therefore, in this quality improvement study, we examined the impact of providing patients with hand hygiene products around mealtime on increasing patient HH adherence and on reducing acquisition of nosocomial antibiotic resistant organisms. **Methods:** Patients on two inpatient leukemia units at a tertiary oncologic center were provided with a single use pre-packaged alcohol wipe on their meal trays prior to every meal (three times daily). Additionally, an information card explaining to patients how and when to use the alcohol wipe was provided on the meal trays three times a week. Both the wipe and instructions were designed with input from patient representatives at the hospital. Two oncologic control units were selected where no specific intervention for patient hand hygiene was conducted. Patient hand hygiene adherence on the control and intervention units were measured through once monthly patient interviews conducted after meals where patients were asked to recall whether they washed their hands prior to eating (using any product). Vancomycin Resistant Enterococcus (VRE) incidence was compared on the intervention and control units during the 7 months prior and 7 months following initiating the intervention. **Results:** During the seven-month intervention period, more than 15 000 wipes were dispensed to patients on the intervention units. Through interview, 91% of 87 patients on the intervention units reported cleaning their hands before eating a meal using any cleaning product compared to 72% of 68 patients on the control units (X2 = 9.32, p = 0.002). Furthermore, on the intervention units, 30 (38%) patients endorsed using the provided hand hygiene product. During intervention period, the combined incidence rate of VRE the intervention units was 1.85 case/1000 patient-days compared to 5.35 cases/1000 patient-days during the 7 months prior to intervention (t = 3.24, p=0.007)(Figure 1). **Conclusions:** This patient-centered quality improvement intervention increased patient hand hygiene and potentially reduced VRE incidence in a vulnerable oncologic population. This practical intervention that incorporated the patient perspective provided accessible hand hygiene products with simple instruction and reminders required minimal participation of unit staff. Further application of the intervention in non-oncologic populations is needed to further establish the relationship between patient hand hygiene and the acquisition of nosocomial infections.

**Figure:**